# Work factors and psychological distress in nurses' aides: a prospective cohort study

**DOI:** 10.1186/1471-2458-6-290

**Published:** 2006-11-28

**Authors:** Willy Eriksen, Kristian Tambs, Stein Knardahl

**Affiliations:** 1 Division of Mental Health, Norwegian Institute of Public Health, Oslo, Norway; 2 Department of General Practice and Community Medicine, University of Oslo, Oslo, Norway; 3 Department of Physiology, National Institute of Occupational Health, Oslo, Norway; 4 Department of Psychology, University of Oslo, Oslo, Norway

## Abstract

**Background:**

Nurses' aides (assistant nurses), the main providers of practical patient care in many countries, are doing both emotional and heavy physical work, and are exposed to frequent social encounters in their job. There is scarce knowledge, though, of how working conditions are related to psychological distress in this occupational group. The aim of this study was to identify work factors that predict the level of psychological distress in nurses' aides.

**Methods:**

The sample of this prospective study comprised 5076 Norwegian nurses' aides, not on leave when they completed a mailed questionnaire in 1999. Of these, 4076 (80.3 %) completed a second questionnaire 15 months later. A wide spectrum of physical, psychological, social, and organisational work factors were measured at baseline. Psychological distress (anxiety and depression) was assessed at baseline and follow-up by the SCL-5, a short version of Hopkins Symptom Checklist-25.

**Results:**

In a linear regression model of the level of psychological distress at follow-up, with baseline level of psychological distress, work factors, and background factors as independent variables, work factors explained 2 % and baseline psychological distress explained 34 % of the variance. Exposures to role conflicts, exposures to threats and violence, working in apartment units for the aged, and changes in the work situation between baseline and follow-up that were reported to result in less support and encouragement were positively associated with the level of psychological distress. Working in psychiatric departments, and changes in the work situation between baseline and follow-up that gave lower work pace were negatively associated with psychological distress.

**Conclusion:**

The study suggests that work factors explain only a modest part of the psychological distress in nurses' aides. Exposures to role conflicts and threats and violence at work may contribute to psychological distress in nurses' aides. It is important that protective measures against violent patients are implemented, and that occupational health officers offer victims of violence appropriate support or therapy. It is also important that health service organisations focus on reducing role conflicts, and that leaders listen to and consider the views of the staff.

## Background

Psychological distress, defined here as anxiety and depression, is a common complaint in Western societies [1]. Nursing personnel, doing both emotional and physical work, and being exposed to psychosocial as well as mechanical stress at work, seem to be one of the occupational groups that are most frequently affected [2].

The relationship between working conditions and the level of psychological distress in employees has been explored in a number of studies. This literature, reviewed a few years ago [3-6], and supplied with new papers during recent years [7-23], shows a consistent association between psychological distress and long working hours, high demands at work, low control at work, low social support at work, and job insecurity. In some studies, psychological distress was also associated with exposure to role ambiguity, interpersonal conflicts, low organisational justice, and bullying, threats, and violence at work.

Still, there is scarce knowledge of how working conditions are related to the level of psychological distress in nursing personnel. Studies have examined nursing students [24], registered (graduate) nurses [25-29], and mixed samples of nursing personnel, hospital employees including nurses, or health-care workers including nurses [10-13,16,20,30-39]. These studies have linked psychological distress to high work demands [12,26,29,30,32,34], low control at work [12,29,32], high job strain [25,29], low social support at work [16,24,25,28,29,34], poor team climate [13], role difficulties and role ambiguity at work [32,34], exposure to threats and violence at work [37,38], exposure to bullying at work [20,39], low organisational justice [11-13], working in male wards [24], working night shifts [10,25,31], inability to quit one's job [34], job insecurity [25], and non-specific occupational stress [31,36]. However, many of these studies were based on small convenience samples, and were, hence, statistically underpowered and potentially unrepresentative. Only some of the studies had a prospective design [12,13,20,24,25], among which we were able to identify two studies based on nursing personnel only [24,25]. No studies seem to have focused on nurses' aides (assistant nurses). Making inferences from studies of other occupational groups may be difficult, as work-demand factors vary from one occupation to another, and because the link between social position and occupational factors may give spurious associations in studies of mixed occupational populations.

The objective of this study was to identify physical, psychological, social, and organisational work factors that predict the level of psychological distress in nurses' aides.

## Methods

### Participants

Nursing personnel in Norway include two large occupational groups: registered nurses and certified nurses' aides. In addition, a smaller group of uncertified nurses' aides have no formal training and often hold temporary jobs. The number of vocationally active nurses' aides was estimated as approximately 55 000 in 1999 (Norwegian Union of Health – and Social Workers, personal communication). About 50 000 of these were members of the Norwegian Union of Health – and Social Workers (the Union).

During the last week of October, 1999, 12 000 nurses' aides were randomly selected from the Union's membership list, and were mailed a questionnaire. After one reminder, 7478 (62.3 %) consented to participate in the study and completed the questionnaire. The membership list also included persons who had retired from working life because of age, disability, or other reasons, and contacts over telephone during the data collection gave the impression that many of these non-working individuals were not motivated to participate in the study. Hence, the true response rate of the vocationally active subjects was probably higher than the overall response rate.

The criteria for inclusion in the present study were: i) being vocationally active and not on leave because of illness or pregnancy at baseline; ii) working more than 18 hours per week, i.e. more than half-time job; and iii) having answered at least three of the five questions about psychological distress. The first criterion was fulfilled by 6485 participants, among whom 5563 fulfilled also the second criterion, and 5076 fulfilled all three criteria. Of these 5076 nurses' aides, 4076 (80.3 %) filled in a second questionnaire and answered at least three of the questions about psychological distress 15 months later.

The reasons that we did not include sick listed individuals in the sample were that many of those who are sick listed at a certain point of time are on a long-term sick leave. There is no information about the length of the sickness absence reported at baseline, but data from Statistics Norway show that more than 60 % of all absence days during a year are part of a sick leave with duration of more than 31 days [40]. Long-term sick leaves could have confounded the results in several ways. Severe health problems as well as the distance in time from last exposure at work could have influenced the reporting of working conditions. Further absence into the observation period could have reduced the exposure to work stress, effects of which we wanted to study.

### Outcome measure

Psychological distress (anxiety and depression) during the previous 14 days was assessed at baseline and follow-up by the SCL-5, a shortened version of the Hopkins Symptom Checklist-25 [41]. The SCL-5 consists of five questions (feeling fearful; feeling hopeless about the future; nervousness or shakiness inside; feeling blue; worrying too much about things), each with four optional answers: not at all (1); a little (2); quite a bit (3); extremely (4). The index was scored as the mean of the item scores. This SCL-5 index has in different studies been shown to correlate strongly (r > 0.90) with the SCL-25 index [41,42], which is a valid measure of psychological distress [43,44]. The internal consistence of SCL-5 is good [41]. Cronbach's alpha in our data is 0.82.

### Measures of working conditions

At baseline, a series of work factors were measured. The practice area in which the aides were working (e.g. nursing home) was recorded, as well as the number of working hours per week (optional answers: 1–9; 10–18; 19–36; > 36), and the frequency of night shift (optional answers: 'never'; 'sometimes'; 'rather often'; 'very often').

Psychological, social, and organisational work factors were measured with questions from the General Nordic Questionnaire for Psychological and Social factors at Work (QPSNordic) [45]. Responses were scored on Likert five-point frequency scales (from '(1) never or very seldom' to '(5) very often or always'), except responses to the question about exposure to bullying, which had only two response options (yes and no) after a precise definition of the concept ('Bullying and harassment, such as badgering, niggling, offending somebody, is a problem at some workplaces and for some workers. To label something bullying, the offensive behaviour has to occur repeatedly over a period of time, and the person confronted has to experience difficulties defending himself/herself. The behaviour is not bullying if two parties of approximately equal 'strength' are in conflict or the incident is an isolated event'). Quantitative work demands were assessed by four questions (work piles up; have to work overtime; have to work at a rapid pace; have too much to do). Positive challenges were assessed by three questions (work is challenging in a positive way; see the work as meaningful; job requires that you acquire new knowledge and skills). Role conflicts were measured with three questions (have to do things that you feel should be done differently; are given assignments without adequate resources; receive incompatible requests from two or more people). Control of work pace was measured with three questions (can set your own work pace; can decide when to take a break; can set your own working hours).  Participation in important decisions was assessed by three questions (can choose which method to use for doing your work; can influence the amount of work; can influence decisions that are important for your work). Social support from immediate superior was assessed by three questions (gives support and help when needed; willing to listen; appreciates your achievements). Fairness of immediate superior's leadership was measured with three questions (distributes the work fairly and impartially; treats the workers fairly and equally; the relationship between you and your superior is a source of stress). Rewards for work well done (money or encouragement) was measured with one question. Feedback about the quality of one's work was also measured with one question. Three aspects of the social climate were assessed (encouraging and supportive; distrustful and suspicious; relaxed and comfortable). Exposure to threats or violence was measured with one question. The work factors that were measured with more than one question (e.g. quantitative work demands) were expressed as indices, calculated as the mean of the item scores. The internal consistency (Cronbach's alpha) of the indices were in the range of 0.68 to 0.88, except the index of control over work pace (0.57).

Exposure to heavy physical work was measured with three questions exploring the frequency of positioning patients manually in the bed, frequency of lifting or supporting patients manually between bed and chair, and frequency of lifting, carrying, or pushing heavy objects, such as heavy furniture and equipment. Optional answers were (in times per shift): 0; 1–4; 5–9; 10 or more. The first two questions were translations of questions developed and validated by British scientists [46]. The extent to which the job required physical endurance was assessed by a question from the QPSNordic, and scored on a Likert five-point frequency scale [45].

At follow-up, the respondents were asked whether they had changed work or work tasks after they completed the first questionnaire. Those who answered 'yes' were asked to mark on a list what kind of consequences this change had had on their work situation. There were eight not mutually exclusive optional answers: more heavy tasks; less heavy tasks; higher work pace; lower work pace; more support and encouragement; less support and encouragement; other consequences; no important consequences.

### Measures of mastery and commitment

Perceived mastery of work and organisational commitment were measured at baseline with questions from the QPSNordic [45]. Organisational commitment was measured with three questions (to my friends I praise this work unit a great place to work; my values are very similar to the work unit's values; this work unit really inspires me to give my very best job). Mastery of work was assessed by four questions (are you content with the quality of the work you do, with the amount of work you get done, with your ability to solve problems at work, with your ability to maintain a good relationship with your coworkers at work?). Responses were scored on Likert five-point frequency scales. The two indices were calculated as the mean of the item scores (Cronbach's alphas: 0.84 and 0.71).

### Measures of background factors

At baseline, a series of factors related to the private sphere were recorded, including age (five-year cohorts), gender, marital status (single vs. married or cohabiting), number of preschool children (0; 1; 2; 3 or more), current pregnancy, and special tasks of care taking during the leisure time, such as caring for handicapped child or old relatives (no; a little; rather much; very much). Physical leisure-time activities in at least one session of 20 minutes per week during the previous three months were also recorded, and were in the present study treated as a dichotomous variable (regular physical exercise vs. no regular exercise or only slow walks). The question about long-term health problems was worded: 'Do you have any kind of long-term or chronic health problem (for instance, asthma, arthritis, chronic pain)? ' Optional answers were: no such problem; yes, but it does not bother me; yes, it bothers me somewhat; yes, it bothers me a lot. The respondents were also asked how many years they had been working as a nurses' aide (< 3 years; 3 – 9 years; > 9 years).

### Statistical analyses

Statistical analyses were conducted with the Statistical Package for Social Sciences (SPSS) version 11.0. Effects of work factors on the level of psychological distress were examined with multivariate, linear regression models.

How to model the effects of exposures on a continuous dependent variable, measured at two points of time, is an unsettled issue that has created an extensive literature over the last three decades [47-49]. We chose to examine effects of work factors on the level of psychological distress by comparing work factors with the follow-up scores of SCL-5, and using baseline scores of SCL-5 as covariate. This method has been recommended instead of comparing exposures with change scores (follow-up scores minus baseline scores), because it controls for baseline imbalances between groups, and has a greater statistical power [48]. Some authors maintain, however, that this method may introduce other biases [49].

The multivariate, linear regression analyses were conducted with all covariates (work factors, baseline psychological distress, and background factors) entered simultaneously. To find the most parsimonious model that still explains the data, and to ensure that effects of some work factors were not obscured by co-linearity, a regression analysis with an automatic stepwise procedure was also conducted.

Practice area represents another level than the other work characteristics, and mastery of work and organisational commitment were considered to be intermediary factors between work characteristics and the outcome measure. These variables were, therefore, analysed separately.

### Ethics

The research protocol was approved by the Committee for Medical Research Ethics. Informed written consent was given by the respondents.

## Results

### Baseline characteristics of respondents and dropouts

Tables 1 and 2 show baseline characteristics of the individuals who responded both at baseline and follow-up (hereafter referred to as respondents). The majority were middle-aged, married or cohabiting women. The level of psychological distress (SCL-5 index) at baseline had a range from 1.00 to 4.00, and the mean (standard deviation) was 1.30 (0.43).

The individuals who dropped out between baseline and follow-up (dropouts) were more likely to be younger than 30, were more likely to be pregnant, were more often single, and had less experience as nurses' aide than the respondents (Table 1). The dropouts reported also a higher baseline level of psychological distress than the respondents (mean difference = 0.06) and a more stressful work situation, including higher quantitative work demands (mean difference = 0.06), more exposure to role conflicts (mean difference = 0.14), less fairness by immediate superior (mean difference = 0.11), poorer social climate (mean difference = 0.09), and more often exposure to bullying at work (7.0 % vs. 3.9 %) than the respondents.

### Changes in the work situation between baseline and follow-up

At follow-up, 630 respondents reported that they had changed work or work tasks after they answered the first questionnaire. The consequences (number of respondents) these changes were reported to have had on the work situation were: 'more heavy tasks' (134), 'less heavy tasks' (279), 'higher work pace' (186), 'lower work pace' (195), 'more support and encouragement' (207), 'less support and encouragement' (59), 'other consequences' (236), 'no important consequences' (62).

### Predictors of the level of psychological distress

The level of psychological distress (SCL-5 index) at follow-up had a range from 1.00 to 4.00, and the mean (standard deviation) was 1.34 (0.49).

Mastery of work and organisational commitment were not significantly related to the level of psychological distress at follow-up, after adjustments for baseline level of psychological distress and background factors (age, gender, marital status, number of preschool children, pregnancy, engagement in special tasks of care taking in the leisure time, regular physical exercise, long-term health problems, and years of experience as nurses' aide) (data not shown).

As shown in Table 3, working in apartment units for the aged was positively associated with the level of psychological distress at follow-up (unstandardised regression coefficient (b) = 0.124; standard error (se) = 0.040; p = 0.002), whereas working in psychiatric departments was negatively associated with the level of psychological distress at follow-up (b = -0.053; se = 0.025; p = 0.036), after adjustments for baseline level of psychological distress, changes in the work situation between baseline and follow-up, and background factors. These associations were somewhat strengthened when specific work factors at baseline (role conflicts etc.) were also entered in the regression model (data not shown).

As shown in Table 4, the level of exposure to role conflicts (b = 0.024; se = 0.011; p = 0.027) and the level of exposure to threats and violence (b = 0.017; se = 0.007; p = 0.011) were positively associated with the level of psychological distress at follow-up, after adjustments for baseline level of psychological distress, other baseline work factors (except practice area), changes in the work situation between baseline and follow-up, and background factors. There was no significant association with changes in the work situation between baseline and follow-up (data not shown). The values of Variance Inflation Factor (VIF) were low (range: 1.05 – 2.57), indicating little problems with co-linearity. The results turned out approximately the same when practice area was also entered in the model (Table 4: model 2b)

Table 5 presents the final equation of the automatic stepwise regression analysis. The level of exposure to role conflicts (b = 0.027; se = 0.009; p = 0.003), the level of exposure to threats and violence (b = 0.016; se = 0.006; p = 0.014), changes in the work situation between baseline and follow-up that resulted in less support and encouragement (b = 0.136; se = 0.055; p = 0.014), being single (b = 0.039; se = 0.017; p = 0.024), the extent to which the respondents were bothered by long-term health problems (b = 0.031; se = 0.007; p < 0.001), and baseline level of psychological distress (b = 0.617; se = 0.016; p < 0.001) were positively associated with the level of psychological distress at follow-up. Changes in work or work tasks between baseline and follow-up that resulted in lower work pace were negatively associated with the level of psychological distress at follow-up (b = -0.123; se = 0.032; p < 0.001).

In a supplementary, stepwise regression analysis, with the three items of the role conflict index entered as separate variables instead of the index, only one of the role conflict items (having to do things that one feels should be done differently) was retained in the final equation (data not shown).

To find out how much of the total variance in the effect variable that the different regression models explained, we examined the adjusted R^2^: In a model with only the baseline level of psychological distress entered as independent variable, the adjusted R^2 ^was 0.340. In a model with baseline level of psychological distress and background factors, the adjusted R^2 ^was 0.342. In a model with baseline level of psychological distress, background factors, all baseline work factors, and all types of changes in the work situation between baseline and follow-up, the adjusted R^2 ^was 0.360. Removing work factors from the full model gave a change in R^2 ^that was statistically significant (p < 0.001).

## Discussion

In this prospective study of Norwegian nurses' aides, most work factors were not predictors of the level of psychological distress. In a linear regression model of the level of psychological distress at follow-up, with baseline level of psychological distress, work factors, and background factors as independent variables, work factors explained 2 % of the variance, whereas the baseline level of psychological distress explained 34 %. The level of exposure to role conflicts, the level of exposure to threats and violence, working in apartment units for the aged, and changes in the work situation between baseline and follow-up that were reported to result in less support and encouragement were positively associated with the level of psychological distress. Working in psychiatric departments, and changes in the work situation between baseline and follow-up that gave lower work pace were negatively associated with psychological distress.

### Comparisons with other studies

There was no difference between respondents working 19–36 hours per week and respondents working more than 36 hours per week with respect to the level of psychological distress 15 months later. Spurgeon et al. concluded that long working hours were a risk factor of mental health disorders, but most of the evidence in their review (up to 1997) was related to situations where working hours exceeded 50 hours per week [6]. Later, studies of nursing personnel showed no difference in the prevalence of psychological distress between those who were working less than 35 hours and those who were working 35 hours or more [25], or between part-time and full-time workers [30].

The frequency of night shifts did not predict psychological distress in the present study. In contrast, earlier studies showed positive associations between night shift work and psychological distress in nurses [10,25] and hospital workers [31]. One study of registered nurses showed no association, though [29].

The frequency of heavy physical work tasks and the extent to which the respondents felt that their work required physical endurance did not predict psychological distress in the present study. Two earlier studies [19,21], both with cross-sectional designs, gave contradictory results – one showed positive association [19] and the other showed inverse association [21] between physical work demands and psychological distress.

The baseline level of quantitative work demands did not predict psychological distress in the present study. On the other hand, changes in the work situation between baseline and follow-up that were reported to have given lower work pace were associated with reduced psychological distress. Many studies have shown an association between high work demands and psychological distress [3,4,9,12,19,26,29,30,32,34], also in nurses or mixed hospital workers [12,26,29,30,32,34], including one with prospective design [12]. No significant association was found in a prospective study of nursing students, though [24].

Exposure to role conflicts at work was positively associated with the level of psychological distress 15 months later. The relationship between role conflicts at work and psychological distress has also been examined in other studies [9,50-52]. However, none of these studies had prospective designs, none focused on nursing personnel, and the results are inconsistent. In a cross-sectional study of hospital workers, psychological distress was linked to "role difficulties", a composite measure that included role conflicts [32].

The level of control over work pace and the level of participation in important decisions at work did not predict psychological distress in the present study. Control at work was found to be inversely associated with psychological distress in many studies [3,4,12,14,19,28,29], also in two cross-sectional studies of registered nurses [28,29] and in a prospective study of hospital workers [12]. Control at work did not predict diagnosed depression, though [13].

The level of support from immediate superior did not predict psychological distress in the present study. On the other hand, changes in the work situation that were reported to have given less support and encouragement at work were associated with increased psychological distress. Social support at work has been found to be inversely associated with psychological distress in many studies [3,4,9,16,24,25,28,29,34,53], also in prospective studies of nursing personnel [24,25]. A prospective study of hospital workers showed no significant association, though [12]. Some studies examined effects of social support from superiors, but with inconsistent results [9,28,33,53].

Feedback about the quality of one's work did not predict psychological distress. Very few other studies, if any, have examined the relationship between feedback about quality of one's work and psychological distress.

Rewards for work well done did not predict psychological distress. Some studies – for example, a prospective study of civil servants [53] – have shown an association between effort-reward imbalance (high efforts combined with low rewards) and psychological distress. Effort-reward balance is not quite the same as the construct used in the present study, though.

The level of fairness in the immediate superior's leadership did not predict psychological distress. In contrast, several other studies showed inverse associations between relational justice (fairness of the supervisor) and psychological distress [11-13,19,22,23], also in hospital workers [11-13], and with prospective design [12,13]. However, these associations were relatively weak, in some studies only seen in women [11,19], in one study only in men [23].

Social climate in the work unit (supportiveness, suspiciousness, and relaxedness) did not predict psychological distress in the present study. In earlier studies, psychological distress was positively associated with tense and prejudiced climate [21], and was negatively associated with coordinated and supportive climate in the work organisation [36]. These were cross-sectional studies, though, and only one focused on nurses [36]. In a prospective study of hospital workers, poor team climate predicted depression [13], but the measure of team climate (participation safety, support for innovation, vision, and task orientation) was very different from the measure of organisational climate used in the present study.

Bullying at work did not predict psychological distress. Other studies, however, have shown association between bullying at work and psychological distress in mixed healthcare personnel [20,39]. One of these studies had prospective design [20].

Frequent exposure to threats and violence at work were positively associated with the level of psychological distress 15 months later. Exposure to threats and violence at work has earlier been linked to psychological distress in nurses or mixed healthcare workers, but only in cross-sectional studies [37,38].

Working in apartment units for the aged was positively associated with psychological distress, whereas working in psychiatric departments was negatively associated with psychological distress. In a prospective study of nursing students, Parkes [24] found increased risk of psychological distress in male wards.

### Explanations of the findings

As expected, exposure to threats and violence predicted psychological distress. Although most violent episodes do not result in serious physical injury, threats and violence are frightening events, and may have long-term psychological consequences, such as easily activated fear of recurring violence [54] and post-traumatic stress disorder.

Exposure to role conflicts predicted psychological distress. Nurses' aides interact with many people at work, including patients, patients' relatives, and professionals, who may all communicate role expectations. Role conflict occurs when role expectations are in conflict, as when the focal person receives incompatible requests from two or more people (intersender role conflict) or incompatible requests from one person (intrasender role conflict), or when there is conflict between the needs or values of the person and the expectations that this person receives from others (person-role conflict) [55]. In the present study, the index was based on three items, each representing one of these types of role conflicts. The analyses showed that it was person-role conflicts (having to do things that one feels should be done differently) that had strongest effects.

The associations with changes in the work situation suggest that it may be possible to influence the level of distress by making changes in working conditions. The fact that variations in psychological distress between practice areas were also seen after adjustments for specific work factors suggests that there are unmeasured work factors of importance, or differential selection into jobs in different practice areas.

The social situation of singles, with more loneliness and less support, could explain the association between being single and psychological distress. The association between long-term health problems and psychological distress confirms what is well known, that health problems may be a psychological stressor.

The most striking finding, however, was the weak association with most of the work factors. For many of these work factors, the values of the coefficients suggested a direction of effects (positive or negative) in line with expectations, but the absolute values of the coefficients in the full regression model were in most cases so low, and the corresponding standard errors were so high, that the findings can hardly be said to represent trends. An exception is the association with the handling of heavy objects, which turned out to be of borderline significance. Handling of heavy material is a well-known risk factor of musculoskeletal disorders, which could, in turn, evoke psychological distress. These effects of material handling may have been underestimated in the present study because of methodological limitations (see below). The association with bullying at work was also of borderline significance, but the value of the coefficient suggested an unexpected inverse relationship, perhaps due to selection bias.

For several work factors, such as quantitative work demands, control at work, and exposure to bullying at work, the results of the present study are inconsistent with the results of earlier studies (see above). This inconsistency with earlier studies, and the fact that work factors explained such a modest part of the level of psychological distress call for an explanation. The question is whether work factors really have so little effects in nurses' aides, or whether the research methods were not able to disclose the effects.

### Methodological considerations

Considering the design of the present study, with adjustments for baseline level of psychological distress, and with an observation period of only 15 months, one would not expect to find strong associations. Adjustment for baseline distress helps to determine the temporal order between exposure and outcome, but it may also, in part, adjust for effects, giving the analyses an element of over-adjustment [49]. If work factors are relatively stable and have affected the level of distress already before baseline, further effects during the following 15 months may be so weak that they could be difficult to uncover. The fact that short-term fluctuations of symptoms between baseline and follow-up were not recorded may have given our design an indirect emphasis on long-term conditions. This may have made it even more difficult to find effects.

The study was based on a large, randomly selected, nationwide sample. The response rate at baseline was not optimal, though. We do not know whether there were differences between the eligible population and the participants, but one should take into account that selection bias due to non-response at baseline could be an important limitation in terms of generalisability. The number of dropouts between baseline and follow-up was low (20 %), but there were several differences between respondents and dropouts with respect to baseline characteristics. Hence, selection bias due to dropout may have influenced the results. A healthy worker selection, due to the fact that vulnerable or unhealthy persons may have avoided specific high-exposure jobs or changed to lower-exposure jobs before entering the study, may have resulted in underestimation of associations between work factors and psychological distress.

The SCL-5 seems to have good validity as measure of psychological distress [41]. The questions used to assess the frequency of patient handling were found to have good validity in a British study [46]. The questionnaire instruments that were used to measure psychological, social, and organisational work factors have been found to have good construct and predictive validity as well as good internal consistency and six-week test-retest reliability [45]. However, the internal consistency of one of the indices (the index of control over work pace) was relatively low (0.57), and the validity of the question used to assess the frequency of handling heavy objects is unknown. Changes in the work situation between baseline and follow-up also represent an uncertainty in our assessment of the work factors. We recorded and adjusted for some types of changes in the work situation, but there may have been types of changes for which we could not control. Besides, information about changes in the work situation between baseline and follow-up was collected at follow-up, and may have been influenced by the respondents' health at this point of time. Hence, the associations between changes of work and psychological distress do not represent prospective relationships from a technical point of view.

The relative homogeneity of the participants in educational attainment and occupation, and the fact that we were able to control for a series of background factors, served to enhance the internal validity of the study. However, the results may have been influenced by background factors for which we were not able to control. Among the potential confounders are work factors other than the ones that were measured, such as predictability at work and job security. Psychological trait factors, such as neuroticism, may also have influenced the results, although much of their confounding effect was probably leveled out by the adjustments for baseline level of psychological distress.

## Conclusion

The study suggests that work factors explain only a modest part of the psychological distress in nurses' aides. Even so, exposure to role conflicts and threats and violence at work may contribute to psychological distress in this occupational group. It is important that protective measures against violent patients are implemented, and that occupational health officers offer victims of violence appropriate support or therapy. It is also important that health service organisations focus on reducing role conflicts, and that leaders listen to and consider the views of the staff. More training may increase nurses' aides' capability to handle conflicting situations, and could be a way to alleviate adverse effects of role conflicts.

## Competing interests

The author(s) declare that they have no competing interests.

## Authors' contributions

WE had the idea, lead the collection of data, designed the study, performed the statistical analyses, and drafted the manuscript. KT helped designing the study, helped interpreting the data, and helped drafting the manuscript. SK helped finding questionnaire instruments, helped designing the study, helped interpreting the data, and helped drafting the manuscript. All authors read and approved the final manuscript.

## Pre-publication history

The pre-publication history for this paper can be accessed here:



## Figures and Tables

**Table 1:** Characteristics of respondents and dropouts.

	Respondents		Dropouts	
Characteristics at baseline	N	%	N	%
Age (years)				
< 30	273	6.7	137	13.7
30–39	824	20.2	221	22.1
40–49	1778	43.6	338	33.8
50–59	1065	26.1	265	26.5
> 59	136	3.3	39	3.9
				
Gender				
Female	3912	96.0	947	94.9
Male	164	4.0	51	5.1
				
Marital status				
Married or cohabiting	3271	80.3	771	77.3
Single	800	19.7	227	22.7
				
Have preschool children				
No	3490	87.0	843	85.8
Yes	522	13.0	140	14.2
				
Special tasks of care taking during leisure time ^†^				
No	2944	72.6	721	72.6
Yes	1112	27.4	272	27.4
				
Pregnant				
No	3978	99.1	955	98.0
Yes	37	0.9	19	2.0
				
Long-term health problems (any kind)				
No or "yes, but not bothered"	3006	74.4	736	74.4
Yes, and bothered by it	1033	25.6	253	25.6
				
Regular physical exercise				
No	1724	42.4	451	45.2
Yes	2343	57.6	547	54.8
				
Years of experience as nurses' aide				
< 10 years	706	17.3	235	23.5
10 years or more	3370	82.7	764	76.5

^† ^Caring for handicapped child, old relatives etc. N = number of individuals. % = column percentages

**Table 2:** Work factors at baseline.

Factor	N	%	Mean	SD	Range
Hours of work per week					
19–36	3512	86.2			
> 36	564	13.8			
Frequency of night shifts					
Never	2075	51.3			
Sometimes	1070	26.5			
Rather often	267	6.6			
Very often	631	15.6			
Positioning patients in bed ^†^					
0	746	19.0			
1–4	1674	42.6			
5–9	971	24.7			
10 or more	537	13.7			
Supporting patients between bed and chair ^†^					
0	683	17.5			
1–4	1737	44.6			
5–9	983	25.2			
10 or more	493	12.7			
Handling heavy objects ^†^					
0	1104	28.5			
1–4	2147	55.5			
5–9	431	11.1			
10 or more	189	4.9			
Exposure to bullying previous 6 months					
No	3905	96.1			
Yes	158	3.9			
Threats and violence			1.76	1.08	1.00 – 5.00
Requirements of physical endurance			3.64	1.16	1.00 – 5.00
Total quantitative work demands			2.89	0.75	1.00 – 5.00
Positive challenges			3.90	0.69	1.00 – 5.00
Role conflicts			2.36	0.80	1.00 – 5.00
Control of work pace			2.36	0.79	1.00 – 5.00
Participation in important decisions			3.33	0.79	1.00 – 5.00
Support from superior			3.85	1.01	1.00 – 5.00
Fairness of leadership			4.12	0.82	1.00 – 5.00
Feedback about quality of work			2.94	1.13	1.00 – 5.00
Rewards for work well done			2.21	1.21	1.00 – 5.00
Social climate			3.99	0.74	1.00 – 5.00

N = Number of respondents in each category. SD = Standard deviation.

^† ^Times per shift.

**Table 3:** The relationship between practice area at baseline and the level of psychological distress at follow-up.

Practice area	N	b (se)
Somatic hospital department	528	- 0.011 (0.019)
Psychiatric hospital department	296	- 0.053 (0.025) *
Paediatric hospital department	71	- 0.052 (0.048)
Nursing home	1705	0.006 (0.013)
Home for the aged	279	0.006 (0.025)
Apartment unit for the aged	102	0.124 (0.040) **
Community nurse unit	659	0.002 (0.017)
Institution for mentally handicapped	417	0.012 (0.021)
Other	259	- 0.023 (0.026)

Results of nine multivariate, linear regression analyses, where persons working in each practice area were compared with persons not working in this area.

N = number of persons in each practice area (the categories are not mutually exclusive, and some persons reported working in two areas).

b (se) = unstardardised regression coefficients (standard error), adjusted for baseline level of psychological distress, changes in the work situation between baseline and follow-up, age, gender, marital status, number of preschool children, pregnancy, the extent of engagement in special tasks of care taking during leisure time, regular physical exercise, the extent the respondents were bothered by long-term health problems of any kind, and years of experience as nurses' aide.

* P < 0.05; ** P < 0.01.

**Table 4:** The relationship between baseline work factors and the level of psychological distress at follow-up. Results of multivariate, linear regression analyses.

Baseline work factor	Model 1 b (se)	Model 2 b (se)	Model 2b b (se)
Hours of work per week ^†^	0.025 (0.018)	0.009 (0.021)	0.006 (0.021)
Frequency of night shifts	0.007 (0.006)	0.005 (0.007)	0.006 (0.007)
Quantitative work demands	0.011 (0.008)	- 0.007 (0.012)	- 0.008 (0.012)
Positioning patients in bed	0.003 (0.007)	- 0.002 (0.011)	0.000 (0.011)
Supporting between bed and chair	0.006 (0.007)	- 0.003 (0.011)	- 0.006 (0.011)
Handling heavy objects	0.022 (0.008) **	0.019 (0.011)	0.020 (0.011)
Requires physical endurance	0.008 (0.005)	0.002 (0.008)	0.000 (0.008)
Positive challenges	- 0.014 (0.009)	- 0.009 (0.011)	- 0.005 (0.011)
Role conflicts	0.027 (0.008) **	0.024 (0.011) *	0.022 (0.011) *
Threats and violence	0.015 (0.006) **	0.017 (0.007) *	0.020 (0.007) **
Participation in decisions	- 0.012 (0.008)	- 0.005 (0.011)	- 0.006 (0.011)
Control of work pace	- 0.010 (0.008)	- 0.008 (0.010)	- 0.009 (0.011)
Support from superior	- 0.008 (0.006)	0.011 (0.011)	0.013 (0.011)
Fairness of leadership	- 0.009 (0.008)	0.001 (0.012)	- 0.001 (0.012)
Feedback about quality of work	- 0.009 (0.006)	- 0.005 (0.008)	- 0.005 (0.008)
Rewards for work well done	- 0.012 (0.005) *	- 0.006 (0.007)	- 0.007 (0.007)
Social climate	- 0.019 (0.009) *	- 0.007 (0.012)	- 0.008 (0.012)
Exposure to bullying ^‡^	- 0.039 (0.033)	- 0.062 (0.035)	- 0.065 (0.035)

* P < 0.05; ** P < 0.01.

b (se) = adjusted, unstardardised regression coefficients (standard error).

Model 1: adjusted for baseline level of psychological distress.

Model 2: adjusted for baseline level of psychological distress, all baseline work factors shown in this table, changes in the work situation between baseline and follow-up, age, gender, marital status, number of preschool children, pregnancy, the extent of engagement in special tasks of care taking during leisure time, regular physical exercise, the extent the respondents were bothered by long-term health problems of any kind, and years of experience as nurses' aide.

Model 2b: adjusted for the same factors as in Model 2, and practice area.

^† ^19 – 36 hours per week = 0; > 36 hours per week = 1.

^‡ ^No = 0; yes = 1.

**Table 5:** Factors related to the level of psychological distress. Final equation in a stepwise, multivariate, linear regression analysis.

Factors	b (se)	β	P
Exposure to role conflicts at work	0.027 (0.009)	0.043	0.003
Exposure to threats and violence	0.016 (0.006)	0.035	0.014
Change in work or work tasks from baseline to follow-up that resulted in lower support and encouragement	0.136 (0.055)	0.034	0.014
Change in work or work tasks from baseline to follow-up that resulted in lower work pace	- 0.123 (0.032)	- 0.054	< 0.001
Marital status ^†^	0.039 (0.017)	0.031	0.024
The extent of being bothered by long-term health problems	0.031 (0.007)	0.063	< 0.001
Baseline level of psychological distress	0.617 (0.016)	0.556	< 0.001

b (se) = adjusted, unstandardised regression coefficients (standard error).

β = adjusted, standardised regression coefficients

The factors not retained in the final equation were: age, gender, pregnancy, number of preschool children, the extent of engagement in special tasks of care taking during leisure time, regular physical exercise, years of experience as nurses' aide, other baseline work factors (except practice area), and other types of changes in the work situation between baseline and follow-up.

^† ^Married or cohabiting = 0; single = 1.
